# India’s neglected tropical diseases

**DOI:** 10.1371/journal.pntd.0006038

**Published:** 2018-03-22

**Authors:** Peter J. Hotez, Ashish Damania

**Affiliations:** 1 Texas Children’s Hospital Center for Vaccine Development, National School of Tropical Medicine, Baylor College of Medicine, Houston, Texas, United States of America; 2 Department of Biology, Baylor University, Waco, Texas, United States of America; Swiss Tropical and Public Health Institute, SWITZERLAND

The Global Burden of Disease Study (GBD) is generating important and often nonintuitive information about the prevalence, incidence, morbidity, and mortality of the world’s major communicable and noncommunicable diseases (NCDs). An emerging narrative from the GBD is the gradual ascendency of the NCDs, especially among the world’s large low- and middle-income countries (LMICs) [1]. But with regards to the neglected tropical diseases (NTDs), we have also seen how these chronic and debilitating infections of poverty might also account for a high percentage of the global disease burden [2].

A more in-depth analysis of NTDs from the GBD 2016 also reveals an important geopolitical dimension of the major NTDs [1]. As shown in Table 1, today the nation of India experiences the world’s largest absolute burden of at least 11 major NTDs. Excluding NTDs that are spatially bound by their requirement for unique insect vectors or snail hosts (e.g., schistosomiasis, onchcocerciasis, human African trypanosomiasis, and Chagas disease), India leads the world in terms of the total number of cases for each of the major NTDs, as defined by the World Health Organization (WHO) [3].

**Table 1:** India’s major NTDs and rank. Data from GBD unless otherwise indicated.

NTDs	Number of cases in India in 2016	Rank globally	Number of cases globally and percentage of cases found in India (in 2016)
Ascariasis	222.2 million	1	799.7 million (28%)
Hookworm disease	102.4 million	1	450.7 million (23%)
Trichuriasis	67.8 million	1	435.1 million (16%)
Dengue^a^	53.2 million	1	101.1 million (53%)
LF	8.7 million	1	29.4 million (29%)
Trachoma^b^	1.8 million	1	3.3 million (53%)
Cysticercosis	819,538	1	2.7 million (31%)
Leprosy (IHME)	187,730	1	523,245 (36%)
Leprosy (WHO)^c^	135,485 new cases; 88,116 prevalent cases	1	New cases 214,783 (63%); prevalent cases 171,948 (51%)
Cystic echinococcosis	119,320	1	973,662 (12%)
Visceral leishmaniasis	13,530	1	30,067 (45%)
Rabies^a^	4,370	1	13,340 (33%)
India’s population in 2016	1.324 billion^d^	2	7.44 billion (18%)

^a^Incident cases

^b^Visual impairment cases only

^c^ [4]

^d^http://databank.worldbank.org/data/

**Abbreviations:** GBD, Global Burden of Disease Study; IHME, Institute for Health Metrics and Evaluation; LF, lymphatic filariasis; NTD, neglected tropical disease.

To be sure, with a population exceeding 1.3 billion, India is the world’s second most populous nation and itself accounts for 18% for the world’s population. Therefore, we might expect that nation to harbor a significant NTD burden. But India also has the seventh largest economy in terms of total gross domestic product (GDP), and several diseases stand out for their disproportionate impact on India’s health. For example, according to the GBD 2016, India accounts for nearly one-half of the world’s prevalent cases of visceral leishmaniasis and one-half of the global incident cases of dengue and visual impairment from trachoma. India also accounts for approximately one-third or more of the prevalent cases of leprosy, lymphatic filariasis (LF), cysticercosis, and incident cases of rabies. However, according to WHO, India may account for more than one-half of the global prevalent and new cases of leprosy [4]. In addition, India also accounts for roughly one-quarter of the world’s ascariasis and hookworm cases. Although no specific information about some other NTDs such as amebiasis or giardiasis are provided, it is possible that India’s high ranking extends beyond the diseases currently considered NTDs by WHO. Moreover, the high-disease-burden NTDs in India are not evenly distributed, but instead focused in areas of urban and rural poverty.

If the global community could focus on India’s NTD problem and make inroads, it would substantially reduce the burden of poverty-related neglected diseases. Therefore, focusing on this single nation could dramatically advance the global health agenda.

In 2017, India’s Prime Minister (PM), Narendra Modi, traveled widely. He visited with the United States president in Washington, DC, became the first Indian PM to visit the nation of Israel, and also visited France, Germany, Russia, and elsewhere [5]. Such meetings present opportunities to discuss the economic advancement of India. Because of the ability of NTDs to reduce worker productivity and child intellectual growth—and ultimately impair India’s economy [6]—these diseases could become a focal point for such discussions.

The good news is that in recent years India has made important progress towards NTD control and elimination. WHO estimates that in 2015, approximately three-quarters of the more than 200 million Indian children that require deworming for their intestinal helminth infections received mass treatment [7]. India is also achieving a similar level of mass treatment coverage for almost 400 million people who are at risk for LF [8], while for years the nation has been committed to multidrug therapy for leprosy (together with rifampicin post-exposure prophylaxis) [9, 10], with such measures resulting in important public health gains. This level of mass drug administration needs to continue or expand in order to reach 100 percent of India’s at-risk or infected population.

Additional opportunities exist to aid India’s progress against many other NTDs, especially vector-borne diseases such as leishmaniasis, dengue, and other arbovirus infections. For example, India hosts a sophisticated and extensive network of biotechnology organizations and private companies capable of developing next-generation vaccines, drugs, and diagnostics. India has the capacity to become a leader in producing next-generation NTD technologies, which could include new leishmaniasis and dengue vaccines and other technologies [11, 12]. To achieve this goal, we’ll need new partnerships between India’s important and mostly private for-profit biotechs and its research universities. One such example is a dengue vaccine partnership between the International Centre for Genetic Engineering and Biotechnology (ICGEB) and Sun Pharma [13]. Together with increased access to these new technologies, a new vision for public–private partnerships would further reduce its NTD burden and indirectly promote economic development.

Presumably due in part to the fact that new NTD technologies are not considered profitable, India has not yet fully organized and directed its powerful biotechnology engine for building tools to combat its autochthonous NTDs. If this dynamic could change, it could produce one of India’s greatest contributions to global health and development [11]. NTD biotechnology for India and the South Asian region should become an important priority for PM Modi’s government. During the 1970s India became a nuclear power. Some 40 years later, the world’s largest democracy now has the opportunity to again flex its scientific and technical muscle to develop cutting-edge NTD technologies.
